# Vitamin D and Its Association with *H. pylori* Prevalence and Eradication: A Comprehensive Review

**DOI:** 10.3390/nu15163549

**Published:** 2023-08-11

**Authors:** Maria Oana Săsăran, Cristina Oana Mărginean, Ancuta Lupu, Ana Maria Koller

**Affiliations:** 1 Department of Pediatrics 3, University of Medicine, Pharmacy, Sciences and Technology George Emil Palade from Târgu Mureș, Gheorghe Marinescu Street No. 38, 540136 Târgu Mureș, Romania; oanam93@yahoo.com; 2 Department of Pediatrics 1, University of Medicine, Pharmacy, Sciences and Technology George Emil Palade from Târgu Mureș, Gheorghe Marinescu Street No. 38, 540136 Târgu Mureș, Romania; 3 Department of Pediatrics, University of Medicine and Pharmacy Gr. T. Popa Iași, Universității Street No. 16, 700115 Iași, Romania; anca_ign@yahoo.com; 4 Clinics of Pediatrics, Emergency County Clinical Hospital, Gheorghe Marinescu Street No. 50, 540136 Târgu Mureș, Romania; kolleranamaria@gmail.com

**Keywords:** vitamin D, deficiency, oral supplements, *H. pylori*, eradication, gastric cancer

## Abstract

Taking into account previous data that sustain a relationship between vitamin D deficiency and higher *H. pylori* infection positivity rates, this review aims to assess the influence of vitamin D deficiency and/or insufficiency upon the prevalence of *H. pylori* infection and its eradication success. Three major databases were searched for articles that analyzed a relationship between vitamin D status and *H. pylori* infection. The literature search retrieved a total of 37 reports, after the article selection process. Hypovitaminosis D emerged as a potential risk factor for *H. pylori* infection, given the higher prevalence of vitamin D deficiency and/or insufficiency among *H. pylori*-positive subjects. Furthermore, the same type of micronutrient deficiency has been directly linked to *H. pylori* eradication failure. An inverse linear relationship between vitamin D status and gastric cancer risk exists, but the additional involvement of *H. pylori* in this correlation is still in question. The potential benefit of oral supplements in enhancing the success of classical therapeutic regimens of *H. pylori* still requires future research. Future population-based studies from larger geographical areas are warranted to address this subject in more depth.

## 1. Introduction

Vitamin D represents one of the essential micronutrients in the human body, the main function of which is to ensure calcium hemostasis and bone mineralization [1]. Available from both exogenous—namely dietary—and endogenous—synthetized after exposure to sunlight—sources, vitamin D is initially present in an inactivated form, either as ergocalciferol or as cholecalciferol [2]. The synthesis of the active form of vitamin D, calcitriol, requires two hydroxylations, which take place in the liver and kidney [3,4]. Calcitriol binds to vitamin D receptors (VDRs), which are found throughout the entire human body and present numerous binding sites as well. Hence, vitamin D regulates multiple biologic processes [3]. The omnipresence of VDRs seems to be one of the key elements through which vitamin D metabolites take part in immune defense. The attachment of pathogen-associated molecular patterns (PAMPs) to toll-like receptors (TLRs), which are activated inside the macrophages as a result of lipopolysacharide (LPS) recognition, triggers an increase in the number of VDRs and consequently enhances the conversion of circulating 25-hydroxyvitamin D into its active form, calcitriol [5,6]. Vitamin D is also involved in T-cell antigen receptor maturation and mediates T-cell immune response [7]. Moreover, 25-hydroxyvitamin D sufficiency is mandatory for normal macrophage function and adequate monocyte production of cathelicidin, an antimicrobial peptide, with an antiviral effect [5,8]. The active involvement of vitamin D in immune processes was certified by multiple studies which successfully proved that there is a link between vitamin D deficiency or insufficiency and the incidence of various infections. Hence, infections of the respiratory and digestive systems and the urinary tract, as well as systemic infections, have been linked to low levels of vitamin D and seem to be prevented by vitamin D supplementation [9,10,11,12,13,14].

*Helicobacter pylori* (*H. pylori*), a spiral-shaped Gram negative bacterium, is ubiquitously spread, infecting more than half of the world’s population [15]. *H. pylori* represents a major causative factor of peptic ulcer disease, an important precursor lesion of gastric cancer, and possesses virulence factors such as cytotoxin-associated gene A (CagA) and vacuolating cytotoxin A, as well as various outer membrane proteins which have been directly linked to the activation of pathways involved in aberrant cell proliferation and gastric carcinogenesis [16,17]. Hence, continuous efforts have been made to eradicate symptomatic *H. pylori* infection, but not without treatment-associated side effects [18]. The increase in antibiotic resistance patterns and *H. pylori* eradication failure represents a threat to human health, which can lead to a higher incidence of gastric cancer [19]. Vitamin D deficiency has been regarded as one of the potential risk factors for *H. pylori* therapeutic failure, with studies proposing its supplementation as an adjuvant to standard medication [20,21]. Moreover, *H. pylori* positivity rates seem to be higher among populations with low serum vitamin D levels [22,23]. Thus, starting from an association between vitamin D deficiency and various types of infections, a link between vitamin D and *H. pylori* infection has been found that constitutes grounds for further research.

This review aims to highlight the association between vitamin D deficiency and/or insufficiency and the prevalence of *H. pylori* infection, its complications, and its eradication success.

## 2. Materials and Methods

Web of Science, Scopus, and PubMed databases were searched for articles published until 15 April 2023 that analyzed a relationship between vitamin D status and *H. pylori* infection. Search terms included “*Helicobacter pylori*” OR “*Helicobacter*” AND “vitamin D” OR “cholecalciferol” OR “calcitriol”. The inclusion criteria were human-based population studies, literature data published in the English language, and research articles that analyzed a correlation between serum vitamin D levels and *H. pylori* incidence and therapeutic management. Papers in-extenso were included, consisting of randomized controlled trials (RCTs), prospective cohort studies, retrospective cross-sectional studies, and longitudinal studies. We excluded studies that did not meet our article objective, case reports, editorials, review articles, and meta-analyses, as well as non-English records, articles without available abstracts, and duplicates/triplicates. Furthermore, abstracts and proceeding papers were excluded from our search results due to the insufficiently detailed information regarding the characteristics of the study population included, diagnostic methods applied for *H. pylori* infection, and the eradication regimen used.

The Rayyan web app aided in the article selection process. Initially, duplicates and triplicates were removed, and afterwards each of the authors examined the records’ titles and their abstracts in order to exclude irrelevant articles. Authors SM and KAM assessed the full-length text of each manuscript to ensure its compliance with inclusion criteria. Potential disagreements between authors were debated and discussed by all authors, who afterwards mutually agreed with the inclusion of each individual record within this review.

## 3. Results

**Figure 1:**
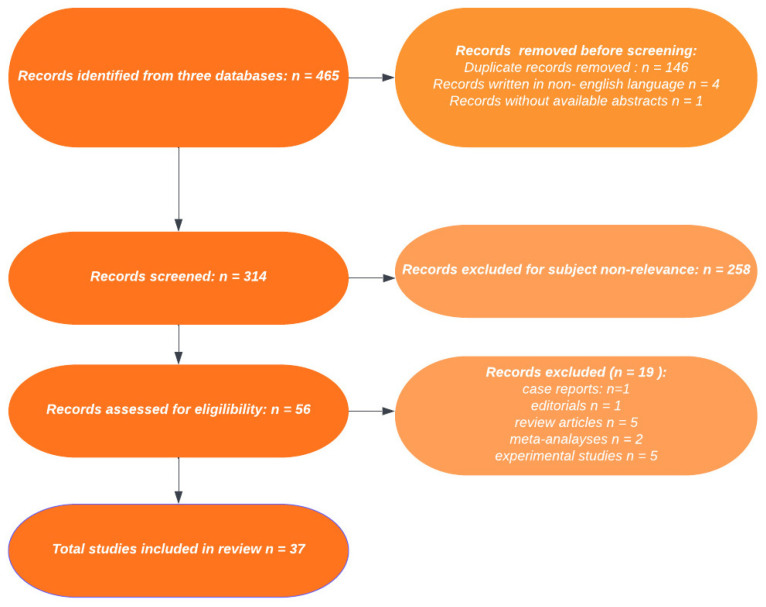
PRISMA flowchart for assessment of eligible studies.

The search retrieved a total of 465 records, which further translated into 319 results after the exclusion of duplicates. The article selection process has been represented in more detail in Figure 1, which complies to the PRISMA 2020 statement and its revised flow diagrams [24]. Four non-English language articles and one article without an available abstract were initially excluded. After exclusion of experimental studies, meta-analyses, review type articles, one case report and one editorial, the final selection pool consisted of 37 articles, the primary or secondary objective of which was to assess a correlation between vitamin D and *H. pylori* infection.

### 3.1. Vitamin D Status: A Possible Risk Factor for H. pylori Infection?

Among various dietary habits, decreased vitamin D intake has emerged as a possible concern related to *H. pylori* infection [25]. Vitamin D deficiency, namely serum levels below 20 ng/mL, poses an increased risk for *H. pylori* infection according to two studies conducted on Turkish and Lebanese adult populations [26,27]. Moreover, an inverse linear correlation was established between vitamin D sufficiency and *H. pylori* infection prevalence, which suggested that adequate vitamin D levels might offer protection against *H. pylori* [26]. Furthermore, one study conducted in Bahrain highlighted that, for each unit decrease in serum vitamin D level, the risk of *H. pylori* infection increases by 1.1 [25]. In a study conducted on an older population with sarcopenia, an inverse relationship between vitamin D deficiency and *H. pylori* was proposed, suggesting that *H. pylori* might actually be the risk factor for vitamin D level decrease [28]. However, Bashir et al. proved on a limited population sample that an 8-week supplementation regimen of high oral vitamin D3 doses can significantly decrease *H. pylori* colonization of the gastric mucosa. Therefore, vitamin D intake is probably influencing the prevalence of this particular infection [29].

Shafrir et al. found an inverse association between vitamin D serum levels and *H. pylori* infection and concluded that those individuals with vitamin D values below 20 ng/mL stand the best chances of acquiring this particular bacterial infection [23]. However, Han et al. reported significantly lower serum vitamin D levels among their study group infected with *H. pylori*, despite including a significant number of patients with levels under 20 ng/mL among the control group as well [30]. Hence, a certain lower limit of serum vitamin D that increases the risk of *H. pylori* infection was put into question. Other authors proposed that vitamin D insufficiency, rather than deficiency, is the major risk factor for *H. pylori* infection [31]. In contrast, one study conducted in Iraq on obese women identified significant differences in terms of *H. pylori* serum antibody positivity only in patients with vitamin D deficiency, while the presence/absence rate of antibodies directed against the same bacterial infection was not influenced by vitamin D insufficiency [32].

Several studies failed to identify an association between serum vitamin D levels and *H. pylori* infection. One study that analyzed the impact of concomitant vitamin D deficiency and *H. pylori* infection upon the prevalence of metabolic syndrome concluded that vitamin D levels were similar among the enrolled individuals, independent of *H. pylori* infectious status. The authors reported that those subjects with both *H. pylori* infection and vitamin D deficiency were the most susceptible to developing metabolic syndrome [33]. Furthermore, among the study participants, who were obese and undergoing bariatric surgery, preoperative nutritional deficiencies such as hypoproteinemia, hypoalbuminemia, hypocalcemia, hypomagnesemia, zinc, copper, folate, or vitamin B12 or vitamin D deficits were encountered independently of *H. pylori* infectious status, confirmed through analysis of gastric mucosa specimens [34]. Contradictorily, another study conducted on obese individuals revealed that vitamin D levels might be higher in the study group infected with *H. pylori*. However, these differences did not reach statistical significance [35].

*H. pylori* co-infection in the setting of other infectious diseases or chronic conditions seems to be additionally associated with vitamin D deficiency. One study conducted on 800 dengue fever patients, divided into two equal groups based on *H. pylori* co-infection status, revealed that those individuals who were positive for the infection presented significantly lower vitamin D serum levels than dengue fever controls [36]. In patients with end-stage renal failure undergoing hemodialysis, a linear positive relationship between vitamin D levels and *H. pylori* immunoglobulin G (IgG) serum antibodies was discovered [37]. Moreover, Bener et al. revealed that, in patients with type 2 diabetes mellitus and high serum titers of *H. pylori* specific antibodies, vitamin D levels were significantly lower than in healthy controls [38]. Similarly, in patients with type 1 diabetes and a positive *H. pylori* stool antigen test, significantly lower values of vitamin D levels were reported [39].

In children, results have been scarce, contradictory, and did not match those obtained in adult populations. Agin et al. found an association between lower vitamin D levels and peptic ulcer in children, but concluded that vitamin D levels did not significantly impact *H. pylori* infection rates within their pediatric study group [40]. Moreover, Urganci et al. concluded that, in children with chronic gastritis, parameters of bone metabolism, such as serum vitamin D, magnesium, calcium, phosphorus or parathormone levels, are similar, regardless of gastro-duodenal *H. pylori* colonization [41]. One study suggested that vitamin D deficiency might represent a risk factor for *H. pylori* infection in infants and toddlers. This study assessed *H. pylori* infection based on serum antibody positivity, which cannot distinguish present from past *H. pylori* infection [42]. Another study, in which a histology based-diagnosis of *H. pylori* was performed, revealed that vitamin D levels are significantly higher in children with with non-*H. pylori* gastro-duodenal conditions, as opposed to infected individuals. Moreover, the same study identified an inverse association between vitamin D levels and CagA positive *H. pylori* strains, which are known to increase gastric cancer risk even further [43]. This finding was not confirmed within the general population of an adult study subgroup who took part in the National Health and Nutrition Examination Survey (NHANES) III, which described a lack of correlation between vitamin D status and CagA seropositivity. However, when separately analyzing a population sample of non-Hispanic whites, the authors identified the same vitamin D deficiency-Cag A seropositivity association as in the aforementioned pediatric study [44]. The studies described within this chapter, which analyzed the relationship between vitamin D serum levels and *H. pylori*, have been depicted in Table 1.

### 3.2. Vitamin D and Its Influence on H. pylori Eradication

Vitamin D deficiency has been regarded as a risk factor for *H. pylori* eradication failure [20,45]. The studies investigating this topic have been summarized in Table 2. Differences in the eradication rates of *H. pylori* positive patients have been linked to vitamin D sufficiency, which translated into serum levels exceeding at least 20 ng/mL. Significantly lower mean serum vitamin D levels have been reported among patients with therapeutic failure [45]. Through their study, Shatla et al. also emphasized that vitamin D sufficiency is one of the key factors for *H. pylori* eradication success [20]. This theory was tested using a classical clarithromycin-based triple eradication therapy of 14 days duration [20]. Magsi et al. also reported an even higher, ascending discrepancy in vitamin D levels between *H. pylori* infected patients that responded to the same therapeutic regimen and those who did not [46]. Similar findings were reported by Almani et al. in their study, in which the identical, classical therapeutic scheme failed to eradicate *H. pylori* infection in more than 80% of patients with vitamin D levels below 30 ng/mL [47]. However, even when considering a modified, novel therapeutic approach consisting of bismuth potassium citrate, proton pump inhibitors (PPIs), amoxicillin, tetracycline, furazolidone, or levofloxacin, vitamin D levels under 20 ng/mL qualified as an independent risk factor for *H. pylori* eradication failure [48]. Therefore, the therapeutic hypothesis of adding vitamin D3 in conjunction with classical eradication schemes emerged. El Shahawy et al. are the first to support the addition of oral vitamin D_3_ supplements to a classical regimen consisting of PPPIs, amoxicillin, and clarithromycin, and to report superior eradication rates of *H. pylori* [49].

A lower vitamin D serum level of 10 ng/mL was proposed as referential for its impact on *H. pylori* eradication. In the study of Yildirim et al., over 80% of the patients in whom *H. pylori* eradication attempt was unsuccessful had serum vitamin D levels below 10 ng/mL [21]. Another study confirmed the very low *H. pylori* eradication rates in patients with vitamin D levels under 10 ng/mL and the best therapeutic success rates for populations with vitamin D serum values ranging between 30 and 34.9 ng/mL [23]. This 10 ng/mL threshold might represent the lowest necessary vitamin D serum level for *H. pylori* eradication success [30].

As previously described, lower vitamin D levels have been found in patients with diabetes and *H. pylori* infection/positive serum antibodies [38,39]. However, Huang et al. were the only ones who evaluated how vitamin D deficiency interferes with *H. pylori* therapeutic success in patients with type 2 diabetes mellitus. Vitamin D levels under 20 ng/mL significantly negatively impacted *H. pylori* eradication, whereas levels ranging between 20 and 30 ng/mL did not influence therapeutic success. Furthermore, the authors acknowledged that vitamin D deficit might also have enhanced dyslipidemia in their study cohort, which, together with prolonged evolution of the glycemic imbalance, could have also contributed to eradication failure [50].

In children, data on the role of vitamin D in *H. pylori* eradication rates are currently limited to a few studies. Zhang et al. found the lowest vitamin D serum values among a pediatric population with *H. pylori* recurrence, despite adequate treatment. Within their study, significantly higher values of the same parameter were found in those patients in whom bacterial eradication was successful and in healthy controls, but each of the analyzed study groups had a low percentage of vitamin D-deficient and -insufficient individuals [51]. Sorokman et al. found that vitamin D levels of over 20 ng/mL are more likely to ensure eradication success. Their study provided no information related to vitamin D supplementation amount and status, which is a routine approach at pediatric ages [43]. In fact, literature data regarding vitamin D intake/supplementation and *H. pylori* eradication success are still very limited. Only one population-based study has managed so far to identify a correlation between a higher vitamin D intake, positively correlated with a higher dietary fish intake, and efficacy of *H. pylori* eradication [52].

### 3.3. VDR and H. pylori Infection

H. pylori induces increased expression of VDRs, which have been shown to play an important role in innate immunity and to render antimicrobial activity. One study conducted on adult patients showed that cells of gastric mucosa specimens harvested from H. pylori-infected patients presented a significant elevation in VDR expression, as opposed to healthy controls [53]. Starting from the theory of possible interference of VDRs with *H. pylori* infection, recent research tried to evaluate the impact of VDR gene’s polymorphisms as well. Martins et al. found a differential distribution of BsmI genotypes of the VDR gene within a study population, divided based on *H. pylori* positivity within gastric mucosa biopsy specimens. The other three polymorphisms studied—namely FokI, ApaI, and TaqI—presented no significant genotype distribution variation among the two study groups [54]. However, in another study, a significant correlation between the Fokl and Apal polymorphisms of the VDR gene and *H. pylori* infection was found [55].

### 3.4. Vitamin D and Gastric Cancer

Vitamin D intake seems to decrease gastric cancer risk, as reflected through a study conducted on a Vietnamese population. The same study also revealed that vitamin D intake is inversely associated with both positive and negative IgG serum antibody titers directed against *H. pylori* [56]. Thus, this study concluded that vitamin D supplementation can protect against gastric cancer, but failed to establish a relationship between daily vitamin D oral intake and *H. pylori* infection [56]. The possible link between hypovitaminosis D and increased cancer risk is further strengthened by Antico et al., who comparatively assessed vitamin D status between four different study groups: one group with atrophic gastritis type A, one group with *H. pylori* gastritis, a group of patients diagnosed with non-specific lymphocytic gastritis, and a control group of healthy subjects. The study group of patients with atrophic, autoimmune-induced gastritis presented the lowest vitamin D levels, followed by the *H. pylori* gastritis group, which also exhibited significantly lower values of serum vitamin D than the other two study groups [57]. Contrary to previous findings, other authors claimed that a higher intake of vitamin D accounts for an increase in gastric cancer risk, even in patients without serological traces of *H. pylori* infection [58].

## 4. Discussion

One meta-analysis of 48 studies pointed out that *H. pylori* infection has been associated with a decrease in serum levels of various vitamins, including vitamin B12, vitamin C, and vitamin D. Higher serum vitamin D and vitamin B12 levels were warranted to improve *H. pylori* eradication rates, whereas vitamin C supplementation helped achieve the same result [59]. This meta-analysis revealed some shortcomings of the analyzed studies, which included limited geographical research areas—as a significant number of studies were conducted on Turkish populations—as well as controversy surrounding adequate vitamin D supplementation [59]. One other meta-analysis, limited to 10 articles, only focused on the impact of vitamin D status upon *H. pylori* infection. Despite the heterogenicity of the included studies, the authors concluded that lower vitamin D levels might account for a higher prevalence of *H. pylori* infection and might negatively influence bacterial eradication [22]. Similar to this meta-analysis, our review included only those studies that analyzed the manner in which vitamin D impacts *H. pylori* infection occurrence and its subsequent eradication. As seen in Table 1 and Table 2, and further detailed in the results section, most of the clinical studies included in this review confirm an inverse relationship between serum vitamin D levels and *H. pylori* infection and eradication rates. However, despite the important number of relevant studies on this topic, their heterogeneity cannot be neglected and hinders the performance of a meta-analysis. Thus, *H. pylori* detection methods vary greatly, whereas the data were mostly limited to Asian studies, which might significantly impact the interpretation of results due to the miscellaneous dietary and nutrition patterns across the globe. Moreover, a handful of studies found no significant relationship between vitamin D and *H. pylori,* and very few of the studies included in this review assessed the importance of vitamin D supplementation [33,34,35,49,52]. The main population benefitting from regular vitamin D supplementation belongs to the pediatric segment. However, the limited number of pediatric studies performed on this subject did not offer any information regarding the regularity, amount, and/or adequacy of the intake of vitamin D supplements within the analyzed study populations [40,41,42,43,51]. Hence, future prospective studies and randomized controlled trials are required to clarify the relationship between vitamin D serum levels, its supplementation, and *H. pylori,* and to evaluate whether the addition of vitamin D3 to therapeutic regimens influences bacterial eradication. Cofounding factors such as age, interfering in the relationship between vitamin D supplementation and *H. pylori* prevalence and eradication, are also subject to future debate, given the lack of regular vitamin D intake among adult populations and the possible inverse relationship between *H. pylori* and vitamin D deficiency, as proposed by Bahși et al. [28].

Our review excluded experimental data, due to the significant abundance of clinical data and the limited number of studies that included animal populations. However, some important aspects can be retained from experimental studies as well, which might aid future clinical research. After artificial infection of the stomach with *H. pylori* in mice, the administration of oral vitamin D3 produced a significant decrease in colonization rates, as well as an upregulation of VDRs [60,61]. The mouse model proved that anti-*H. pylori* activity of vitamin D3 is further enhanced by the activation of the VDR-Cathelicidin antimicrobial peptide (CAMP) pathway [60,61]. Another in vitro study showed that vitamin D3 supplementation triggers a restoration of lysosomal degradation through the activation of the protein disulfide-isomerase A3 (PDIA3) receptor, which promotes calcium release from lysosomes, lysosomal acidification and, consequently, the elimination of *H. pylori* through the autolysosomal pathway [62]. Furthermore, the synthetical production of indene compounds derived from vitamin D led to selective antibacterial action against *H. pylori* [63,64]. More specifically, vitamin D decomposition products, such as vitamin D3 decomposition product 1 (VDP1), detain an anti-*H. pylori* action through induction of the bacteria’s cell membrane structure collapse [65]. A synergistic effect of the addition of 1α, 25-dihydroxyvitamin D3 to the standard quadruple therapy in the eradication of *H. pylori* has been proposed, after the compound was proven to protect against *H.pylori*-induced apoptosis of gastric epithelial cells [66]. This theory is further supported by the important tumor suppressor role of the vitamin D3 upregulated protein 1 (VDUP1), which was shown to protect against gastric carcinogenesis [67]. Thus, in light of these recently surfaced experimental data, future population-based studies are required to shine a light upon the role of vitamin D3 supplementation in preventing *H. pylori* infection and in facilitating its eradication. Pediatric studies could provide more insight into this matter, given that vitamin D supplements are routinely administered in children [68]. The data available so far for pediatric ages did not provide any information regarding the amount of vitamin D supplementation that the enrolled populations were receiving [40,41,42,43,51].

The link between vitamin D intake and gastric cancer risk, while taking into consideration the presence/absence of *H. pylori* infection, still requires more research. Two review articles clearly underlined an inverse correlation between vitamin D serum levels and gastric cancer risk, morbidity, and mortality, but most of the studies included in these articles did not perform a separate analysis on *H. pylori* infection status [69,70]. As previously reported in the results section, patients with gastric cancer and atrophic gastritis, infected with *H. pylori,* might present lower vitamin D serum levels, but research on this matter needs to be expanded [4,57]. Furthermore, the clear mechanisms through which vitamin D offers protection against gastric cancer, possibly through VDR interaction, still require elucidation [69,70].

A few previous reviews also provided some important take-home messages on the role of vitamin D in *H. pylori* infection. The bacteriolytic action of hydrophobic lipid structures, such as indene compounds, vitamin D, and 3-carbonyl steroids, has been recently highlighted [71]. Among multiple micronutrients that can influence *H. pylori*’s survival, pathogenicity, and eradication, vitamin D distinguished itself as an important regulator of host immune response [72]. Hence, one literature survey (Golpour et al.) proposed potential antibiotic-independent approaches for *H. pylori* treatment based on vitamin D supplements [73]. However, these review articles had a narrative structure and referred to fewer population-based studies than the present work.

## 5. Conclusions

This comprehensive review highlights the impact of vitamin D deficiency and/or insufficiency upon an ascending prevalence of *H. pylori* infection and its eradication failure. Vitamin D-deficient subjects might be more prone to developing *H. pylori* infection, but the influence of *H. pylori* infection upon vitamin D status is still puzzling. The role of vitamin D intake is still in question, as most of the reviewed studies failed to analyze whether the addition of oral supplements influences the performance of current therapeutic regimens. Thus, the screening of vitamin D levels in subjects with *H. pylori* infection might establish the need for its supplementation in ensuring therapeutic success. Vitamin D deficit might be connected to an increase in gastric cancer risk, but the additional influence of *H. pylori* in this linear relationship is still unclear. Furthermore, the currently available data are hindered by the enrollment of populations from restricted geographical regions, as well as by the heterogenicity of *H. pylori* infection detection methods and the reduced number of pediatric populations included so far. Future population-based studies and randomized clinical trials from larger geographical areas are warranted to address this subject in more depth and to analyze the synergistic effect of vitamin D supplementation and classical therapeutic schemes.

## Figures and Tables

**Table 1:** Characteristics of clinical studies that assessed the impact of vitamin D deficiency upon *H. pylori* infection.

Reference (Author, Year)	Type of Study	Population and Study Group Assignment	*H. pylori* Detection Method	Main Outcome
Habbash et al., 2022 [25]	Retrospective cross-sectional study	200 adults patients: 111 *H. pylori* positive patients 89 *H. pylori* negative patients	Gastric biopsy microscopic evaluation in 51% of the study population Urea breath testing in 35.5% of the study population Both testing methods in 13.5% of the study population	Significantly higher prevalence of *H. pylori* infection among participants with vitamin D deficiency
Mut Surmeli et al., 2019 [26]	Retrospective cross-sectional study	254 adult patients: 43 *H. pylori* positive patients 211 *H. pylori* negative patients	Histopathological examination of gastric biopsy specimens	Median serum vitamin D levels were significantly lower in the *H. pylori* positive group, when compared to *H. pylori* negative group Vitamin D deficiency (<20 ng/mL) was significantly more frequent in *H. pylori* positive group There was an inverse linear relationship between serum level of vitamin D and *H. pylori* positivity
Assaad et al., 2018 [27]	Cross-sectional study	294 adult patients: 154 *H. pylori* positive patients 140 *H. pylori* negative patients	Histopathological examination of gastric biopsy specimens	Significantly higher prevalence of vitamin D serum level < 20 ng/mL among the *H. pylori* positive study group
Bahşi et al., 2022 [28]	Retrospective cross-sectional study	101 adult patients: 27 patients with sarcopenia 74 patients without sarcopenia	Histopathological examination of gastric biopsy specimens	Significantly lower vitamin D serum levels among patients with sarcopenia and *H. pylori* infection Significantly higher prevalence of vitamin D serum level < 20 ng/mL among patients with sarcopenia and *H. pylori* infection
Bashir et al., 2016 [29]	Interventional monocentric open pilot study	15 healthy adults: 3 patients diagnosed with *H. pylori* infection	Histopathological examination of gastric biopsy specimens	In the subgroup diagnosed with *H. pylori* infection, an 8-week supplementation regimen of high oral vitamin D_3_ doses led to a significant decrease in *H. pylori* colonization of the gastric mucosa
Shafrir et al., 2021 [23]	Retrospective cross-sectional study	150,483 adult patients: 75,640 *H. pylori* positive patients 74,843 *H. pylori* negative patients	Urea breath test in 99% of cases Stool antigen test in the rest of the cases	Mean serum vitamin D levels were significantly higher in the *H. pylori* negative group There was an inverse linear relationship between serum level of vitamin D and *H. pylori* positivity Higher prevalence of *H. pylori* among individuals with vitamin D values < 20 ng/mL
Han et al., 2019, [30]	Multicenter, observational and prospective cohort study	672 adult patients: 415 *H. pylori* positive patients 257 *H. pylori* negative patients	Urea breath test	Significantly lower serum vitamin D levels in the *H. pylori* positive group
Assaad et al., 2019 [31]	Retrospective cross-sectional study	460 adult patients: 225 *H. pylori* positive patients 235 *H. pylori* negative patients	Histopathological examination of gastric biopsy specimens	Significantly higher prevalence of vitamin D insufficiency among the *H. pylori* positive study group An inverse relationship was found between two single nucleotide polymorphisms of TLR4 and vitamin D levels
Mohammed et al., 2021 [32]	Retrospective cross-sectional study	100 healthy obese women: Group 1–3 patients with vitamin D insufficiency and *H. pylori* infection Group 2–10 patients with vitamin D insufficiency and negative for *H. pylori* Group 3–13 patients with vitamin D deficiency and *H. pylori* infection Group 4–74 patients with vitamin D deficiency and negative for *H. pylori*	Detection of specific serum antibodies (IgM and IgG) directed against *H. pylori*	Differences in terms of *H. pylori* serum antibody positivity were seen only between groups 3 and 4, which were vitamin D deficient No differences in antibody positivity rate between individuals with vitamin D insufficiency
Chen et al., 2016 [33]	Prospective community-based study	2113 adult patients: 557 patients with metabolic syndrome 1556 patients without metabolic syndrome	Urea breath test	Vitamin D levels were similar among the enrolled population, independently of *H. pylori* infectious status Subjects infected with *H. pylori* and vitamin D deficiency were the most susceptible to developing metabolic syndrome
Gerig et al., 2013 [34]	Retrospective cross-sectional study	404 obese adult patients undergoing bariatric surgery: 85 *H. pylori* positive patients 319 *H. pylori* negative patients	Histopathological examination of gastric biopsy specimens	There were no significant differences in vitamin D levels between the two study groups
Mihalache et al., 2016 [35]	Cross-sectional, observational study	93 obese adult patients: 47 *H. pylori* positive patients 46 *H. pylori* negative patients	Histopathological examination of gastric biopsy specimens	Slightly higher mean serum levels of vitamin D were found among patients infected with *H. pylori* infection, but this difference did not reach statistical significance
Mirza et al., 2022 [36]	Prospective case-control study	800 adult subjects: 400 patients with dengue fever (*H. pylori* positive and *H. pylori* negative) 400 healthy controls (*H. pylori* positive and *H. pylori* negative)	Urea breath test	Patients with dengue fever and *H. pylori* co-infection exhibited significantly lower vitamin D levels than healthy controls diagnosed with *H. pylori* infection Dengue cases co-infected with *H. pylori* presented higher chances of having vitamin D deficiencies than those in whom the bacterial infection was not detected
Nasri et al., 2007 [37]	Cross-sectional study	36 adult patients with end-stage renal disease undergoing hemodialysis	Detection of specific serum antibodies (IgG) directed against *H. pylori*	Significant positive association between vitamin D serum levels and *H. pylori* specific IgG antibody titer
Bener et al., 2020 [38]	Prospective case-control study	1058 adult subjects: 529 patients with type 2 diabetes mellitus 529 healthy age-matched controls	Detection of specific serum antibodies (IgA and IgG) directed against *H. pylori*	Significantly lower vitamin D serum levels among subjects with type 2 diabetes mellitus and IgA/IgG seropositivity as opposed to controls with positive serum antibodies
Zawada et al., 2023 [39]	Cross-sectional study	103 adult patients with type 1 diabetes mellitus: 31 *H. pylori* positive patients 72 *H. pylori* negative patients	*H. pylori* stool antigen test	Significantly lower vitamin D serum levels among subjects with *H. pylori* infection Significant association between lower vitamin D serum levels and positive *H. pylori* stool antigen tests
Agin et al., 2021 [40]	Prospective case-control study	291 children who underwent an upper digestive endoscopy: 38 patients with peptic ulcer 253 patients without peptic ulcer	Histopathological examination of gastric biopsy specimens	Significant association between lower serum vitamin D levels and the presence of peptic ulcer No variations in vitamin D levels in relation to *H. pylori* infection
Urganci et al., 2020 [41]	Prospective cross-sectional study	100 children with chronic gastritis: 72 *H. pylori* positive patients 28 *H. pylori* negative patients	At least two of three examinations: Histopathological examination of gastric biopsy specimens Culture of gastric biopsy specimens Rapid urease test of gastric biopsy specimens	Serum vitamin D levels were similar between the two study groups
Gao et al., 2020 [42]	Retrospective cross-sectional study	6896 apparently healthy infants and toddlers: 2113 *H. pylori* seropositive individuals 4783 *H. pylori* seronegative individuals	Detection of specific serum antibodies (IgG) directed against *H. pylori*	Significantly higher prevalence of vitamin D deficiency among the *H. pylori* seropositive children
Sorokman et al., 2020 [43]	Prospective observational cohort study	128 children with duodenal ulcer and chronic gastro-duodenitis: 86 *H. pylori* positive patients 42 *H. pylori* negative patients	Histopathological examination of gastric biopsy specimens	Significantly lower vitamin D serum levels among subjects with *H. pylori* infection Inverse association between vitamin D levels and cytotoxin-associated gene A (CagA) positive *H. pylori* strains
Kuang et al., 2022 [44]	Cross-sectional study	3512 *H. pylori* seropositive *H. pylori* patients: 1412 *H. pylori* CagA seropositive subjects 2100 *H. pylori* CagA seronegative subjects	Detection of specific serum antibodies (IgG) directed against *H. pylori* CagA antigen	Inverse association between vitamin D levels and cytotoxin-associated gene A (CagA) positive *H. pylori* strains among the non-Hispanic white population

Legend: CagA—cytotoxin-associated gene A; *H. pylori*—*Helicobacter pylori*, Ig—immunoglobulin, TLR4—tool like receptor 4.

**Table 2:** Characteristics of clinical studies that assessed the impact of vitamin D deficiency upon *H. pylori* eradication.

Reference (Author, Year)	Type of Study	Population and Study Group Assignment	*H. pylori* Eradication Scheme	Main Outcome
Shatla et al., 2021 [20]	Longitudinal study	150 adults infected with *H. pylori*: group I—108 patients in whom *H. pylori* eradication was successful group II—42 patients with *H. pylori* eradication failure	Clarithromycin-based triple eradication therapy of 14 days	Mean serum levels of vitamin D were higher in group I Percentage of patients with vitamin D deficiency was significantly higher in group II
El Shahawy et al., 2018 [45]	Longitudinal study	150 adults with *H. pylori* chronic gastritis: group I—110 patients with vitamin D sufficiency (serum levels > 20 ng/mL) group II—40 patients with vitamin D deficiency (serum levels < 20 ng/mL)	Clarithromycin-based triple eradication therapy of 14 days	Significantly lower mean serum vitamin D levels in the eradication failure group compared to the successful treatment group Prevalence of vitamin D deficiency was significantly higher in the failed treatment group
Magsi et al., 2021 [46]	Longitudinal study	128 adults with *H. pylori* gastritis: 92 patients in whom *H. pylori* eradication was successful 36 patients with *H. pylori* eradication failure	Clarithromycin-based triple eradication therapy of 14 days	Significantly lower mean serum vitamin D levels in the eradication failure group compared to the successful treatment group Prevalence of hypovitaminosis D was significantly higher in the eradication failure group
Almani et al., 2020 [47]	Retrospective study	100 adults with *H. pylori* gastritis: 62 patients in whom *H. pylori* eradication was successful 38 patients with *H. pylori* eradication failure	Clarithromycin-based triple eradication therapy of 14 days	Significantly lower mean serum vitamin D levels in the eradication failure group compared to the successful treatment group Prevalence of vitamin D deficiency was significantly higher in the eradication failure group
Lan et al., 2022 [48]	Prospective cohort study	341 adults with *H. pylori* infection: 273 patients in whom *H. pylori* eradication was successful 68 patients with *H. pylori* eradication failure	Bismuth potassium citrate + PPI + amoxicillin + Tetracycline/Levofloxacin	Vitamin D levels under 20 ng/mL represented an independent risk factor for *H. pylori* eradication failure
El Shahawy et al., 2021 [49]	RCT	150 adult patients with dyspeptic symptoms and *H. pylori* infection: Group A—75 patients who received clarithromycin-based triple eradication therapy Group B—75 patients who received vitamin D3 + clarithromycin-based triple eradication therapy	Group A—clarithromycin-based triple eradication therapy of 14 days Group B—vitamin D3 for 1 month + clarithromycin-based triple eradication therapy of 14 days	Significantly higher *H. pylori* eradication rates in group B
Yildirim et al., 2017 [21]	Prospective observational study	220 adults with *H. pylori* infection: 170 patients in whom *H. pylori* eradication was successful 50 patients with *H. pylori* eradication failure	Colloidal bismuth sub-citrate + Pantoprazole + Tetracycline + Metronidazole	Significantly lower mean serum vitamin D levels in the eradication failure group compared to the successful treatment group Prevalence of vitamin D < 10 ng/mL was significantly higher in the eradication failure group
Shafrir et al., 2021 [23]	Retrospective cross-sectional study	150,483 adult patients: 75,640 *H. pylori* positive patients 74,843 *H. pylori* negative patients	Bismuth-based quadruple therapy/Clarithromycin-based triple therapy/Clarithromycin-based quadruple therapy/Triple therapy (PPI/Bismuth + 2 of the following antibiotics: Amoxicillin, Metronidazole and Tetracycline) for 14 days	Significantly lower mean serum vitamin D levels in the eradication failure group compared to the successful treatment group
Han et al., 2019 [30]	Multicenter, observational and prospective cohort study	672 adult patients: 415 *H. pylori* positive patients 257 *H. pylori* negative patients	Amoxicillin + Clarithromycin + Colloidal bismuth tartrate + Esomeprazole/Amoxicillin + Clarithromycin + Colloidal bismuth tartrate + Rabeprazole for 14 days	A serum vitamin D level of at least 10 ng/mL was independently associated with *H. pylori* eradication success
Huang et al., 2019 [50]	Retrospective observational study	160 adult patients with type 2 Diabetes mellitus and *H. pylori* infection: 124 patients in whom *H. pylori* eradication was successful 36 patients with *H. pylori* eradication failure	Amoxicillin + Clarithromycin + Esomeprazole + Bismuth potassium citrate for 14 days	Significantly lower mean serum vitamin D levels in the eradication failure group compared to the successful treatment group Serum vitamin D deficiency (<20 ng/mL) was a significant risk factor for *H. pylori* eradication failure
Zhang et al., 2020 [51]	Longitudinal study	142 pediatric patients with *H. pylori* infection: 106 patients in whom *H. pylori* eradication was successful 36 patients with *H. pylori* eradication failure	Clarithromycin-based triple therapy for 14 days	Significantly lower mean serum vitamin D levels in the eradication failure group compared to the successful treatment group Lowest vitamin D serum levels were encountered among patients with *H. pylori* recurrence
Sorokman et al., 2020 [43]	Prospective observational cohort study	128 children with duodenal ulcer and chronic gastro-duodenitis: 86 *H. pylori* positive patients 42 *H. pylori* negative patients	Clarithromycin-based triple therapy for 14 days	Serum vitamin D deficiency (<20 ng/mL) was more prevalent among patients with *H. pylori* eradication failure
Ikezaki et al., 2017 [52]	Prospective observational cohort study	352 adults patients with gastritis and/or duodenal ulcer and *H. pylori* infection: 212 patients in whom *H. pylori* eradication was successful 140 patients with *H. pylori* eradication failure	Clarithromycin-based triple therapy for 14 days	Significantly lower mean vitamin D intake in the eradication failure group compared to the successful treatment group Higher vitamin D intake was positively correlated with higher dietary fish intake

Legend: *H. pylori*—*Helicobacter pylori*, PPI—proton pump inhibitor, RCT—randomized controlled trial.

## Data Availability

No new data were created or analyzed in this study. Data sharing is not applicable to this article.
